# Qushi Huayu Decoction Inhibits Hepatic Lipid Accumulation by Activating AMP-Activated Protein Kinase *In Vivo* and *In Vitro*

**DOI:** 10.1155/2013/184358

**Published:** 2013-03-19

**Authors:** Qin Feng, Xiao-jun Gou, Sheng-xi Meng, Cheng Huang, Yu-quan Zhang, Ya-jun Tang, Wen-jing Wang, Lin Xu, Jing-hua Peng, Yi-yang Hu

**Affiliations:** ^1^Institute of Liver Diseases, Shuguang Hospital Affiliated to Shanghai University of Traditional Chinese Medicine, 258 Zhangheng Road, Pudong District, Shanghai 201203, China; ^2^Drug Discovery Laboratory, School of Pharmacy, Shanghai University of Traditional Chinese Medicine, Shanghai 201203, China; ^3^Experimental Center for Science and Technology, Shanghai University of Traditional Chinese Medicine, Shanghai 201203, China; ^4^Shanghai Key Laboratory of Traditional Chinese Clinical Medicine, Shanghai 201203, China

## Abstract

Qushi Huayu Decoction (QHD), a Chinese herbal formula, has been proven effective on alleviating nonalcoholic fatty liver disease (NAFLD) in human and rats. The present study was conducted to investigate whether QHD could inhibit hepatic lipid accumulation by activating AMP-activated protein kinase (AMPK) *in vivo* and *in vitro*. Nonalcoholic fatty liver (NAFL) model was duplicated with high-fat diet in rats and with free fatty acid (FFA) in L02 cells. In *in vivo* experimental condition, QHD significantly decreased the accumulation of fatty droplets in livers, lowered low-density lipoprotein cholesterol (LDL-c), alanine aminotransferase (ALT), and aspartate aminotransferase (AST) levels in serum. Moreover, QHD supplementation reversed the HFD-induced decrease in the phosphorylation levels of AMPK and acetyl-CoA carboxylase (ACC) and decreased hepatic nuclear protein expression of sterol regulatory element-binding protein-1 (SREBP-1) and carbohydrate-responsive element-binding protein (ChREBP) in the liver. In *in vitro*, QHD-containing serum decreased the cellular TG content and alleviated the accumulation of fatty droplets in L02 cells. QHD supplementation reversed the FFA-induced decrease in the phosphorylation levels of AMPK and ACC and decreased the hepatic nuclear protein expression of SREBP-1 and ChREBP. Overall results suggest that QHD has significant effect on inhibiting hepatic lipid accumulation via AMPK pathway *in vivo* and *in vitro*.

## 1. Introduction 

Nonalcoholic fatty liver disease (NAFLD) is an increasingly common health concern that it is considered to be a component of the metabolic syndrome. Excessive accumulation of triglyceride (TG) in hepatocytes is the key factor in NAFLD. The spectrum of NAFLD can range from simple fatty liver (hepatic steatosis), with a begin prognosis, to the potentially progressive form of nonalcoholic steatohepatitis (NASH), which can lead to fibrosis and cirrhosis [1, 2]. The origin of this disease is unknown and probably multifactorial [3]. Nevertheless, impaired lipid metabolism is recognized as an associate and/or promoting mediator of the disease; management of hepatic metabolic disorders becomes an essential strategy for prevention and treatment of obesity-related NAFLD [4, 5].

AMP-activated protein kinase (AMPK) is a key sensor of cellular energy status and it is also a major regulator of liver and whole body lipid homeostasis [6, 7]. AMPK integrates nutritional and hormonal signals to promote energy balance by switching on catabolic pathways and switching off ATP-consuming pathways, both by short-term effects on phosphorylation of regulatory proteins and by long-term effects on gene expression. Activation of AMPK in the liver leads to the stimulation of fatty acid oxidation and inhibition of lipogenesis, glucose production, and protein synthesis. AMPK activation in the liver results in the phosphorylation and inactivation of acetyl-CoA carboxylase (ACC), a direct AMPK substrate, which leads to decreased conversion of acetyl-CoA to malonyl CoA and decreased fatty acid synthesis [8, 9]. AMPK also decreases fatty acid synthesis by reducing the protein level of sterol regulatory element-binding protein-1c (SREBP-1c) and downregulating carbohydrate-responsive element-binding protein (ChREBP) by phosphorylation of Ser568 under hyperlipidemic conditions, which are two key transcription factors regulating *de novo *lipogenesis [9–11]. Since AMPK plays a major role in the control of hepatic metabolism, it is considered as a therapeutic target for fatty liver disease [12].

Traditional medicines have long been used in China, and most of them are orally administrated. Qushi Huayu Decoction (QHD), a Chinese herbal formula, has a long history of use in clinical practice to alleviate NAFLD [13]. Previous animal studies showed that QHD could ameliorate hepatic steatosis in rat models and HepG2 cells [14]. The decoction is capable of reducing tumor necrosis factor-*α* (TNF-*α*) expression through cathepsin B and the nuclear factor *κ*B pathway in rats with nonalcoholic steatohepatitis and in steatosis HepG2 cells [14, 15]. However, the molecular mechanism involved in the effect on inhibiting hepatic lipid accumulation needs further investigation.

Considering the key role of AMPK activation in the regulation of lipid metabolism and the potential capability of QHD in preventing hepatic steatosis, we hypothesized that AMPK might be a mediator due to the effects of QHD. So in this study, we observed the effect of QHD on AMPK activation and the related pathways in hepatic lipogenesis in high-fat diet-induced obese rats and also in FFA-induced steatosis L02 cells.

## 2. Materials and Methods

### 2.1. Antibodies

For western blot analysis, polyclonal antibodies of AMPK*α*, phospho-Thr172-AMPK*α*, ACC*α*, and phospho-Ser79-ACC*α* were obtained from Cell Signaling Technology, USA. Polyclonal antibodies of SREBP-1 and ChREBP were obtained from Abcam, USA. Monoclonal antibody of *α*-tublin was obtained from EPITMICS, USA. Polyclonal antibody of Lamin B was obtained from Santa Cruz Biotechnology, USA.

### 2.2. Preparation of QHD

Qushi Huayu Decoction consists of five dried crude herbs listed in Table 1. The ratio of *Herba Artemisiae capillaris, Rhizoma Polygoni cuspidati, Herba Hyperici Japonici, Rhizoma Curcumae longae, *and *Gardenia jasminoides *Ellis is 4 : 3 : 3 : 2 : 2. Herbs were obtained from qualified suppliers and on the basis of standards specified in the Chinese Pharmacopoeia (2010 edition). The herbs were extracted with ethanol or water, condensed to the density of 0.93 g/crude herb/mL, and stored at −20°C until further use. The formula was patented in the State Intellectual Property Office of China (ID: ZL200610009140.0.0).

### 2.3. High-Pressure Liquid Chromatography (HPLC) Analysis of QHD

High performance liquid chromatography was used for analyzing the quality of formula by monitoring the four active components in it. The standards used for the quantitative analysis of QHD were chlorogenic acid, polygonin, resveratrol, and jasminoidin, which are currently recommended for analyzing QHD. These standards were obtained from the Chinese National Centre for Quality Control of Traditional Chinese Medicine, China. The QHD and standard compounds were dissolved in methanol, filtered through 0.2 *μ*m nylon filters, and analyzed in HPLC.

Liquid chromatography was performed on an Agilent 1200 HPLC system (Agilent Technologies) equipped with a quaternary solvent delivery system, online degasser, autosampler, and column temperature controller. A mixture of methanol and water as mobile phase in a gradient mode was used in Edipse XDB-C18 (4.6 mm × 250 mm, 5*μ*m) column. The gas flow was 1.8 L/min with a 1 mL/min flow rate and the column was maintained at 40°C. The detection was done at 254 nm wavelength.

### 2.4. *In Vivo* Experimental Design

Thirty male Sprague-Dawley (SD) rats, weighing 170 ± 20 g, were obtained from Shanghai Experimental Animal Center of Chinese Academy of Sciences, China. They were maintained in a temperature-controlled room (25 ± 1°C on a 12 h : 12 h light-dark cycle) in the animal center (Shanghai University of Traditional Chinese Medicine, Shanghai, China). The study was carried out under the guidelines for animal experimentation set by them, and the protocol was approved by the animal studies ethics committee of Shanghai University of TCM.

After a week acclimation, thirty rats were randomly divided into three groups of 10 rats each. One group (normal diet, ND, *n* = 10) of rats were fed with 13.8% kcal fat diet (Shanghai Laboratory Animal, Shanghai, China; protein: 27.5%, carbohydrate: 60.5%, and fat: 13.8% kcal/g), and two groups (high-fat diet, HFD, *n* = 10; high-fat diet along with QHD, HFD + QHD, *n* = 10) were fed with 36.5% kcal fat diet (Shanghai Laboratory Animal, Shanghai, China; protein: 18.9%, carbohydrate: 44.6%, and fat: 36.5% kcal/g). Food and water were available ad libitum. After 4 weeks, rats of HFD + QHD group were dosed by oral gavage once per day for 4 weeks with QHD of 0.1 mL/kg·d. At the end of the 8th week, that is, QHD treatment remained for 4 weeks, all the animals from three groups were sacrificed and the liver was removed and stored at −70°C for subsequent analysis.

### 2.5. Measurement of Triglyceride (TG) and Free Fatty Acid (FFA) Content in Liver

The wet liver weighing 200 mg was homogenized in 3 mL of ethanol-acetone (1 : 1) and kept at 4°C for 12 hours. Afterward, the sample was centrifuged at 3000 rpm for 15 minutes and the suspension was collected for the determination of triglyceride content by using biochemistry assay kits (Dongou Biology Technique Co. Ltd., Zhejiang, China). The amount of TG in the liver was expressed as mg/g wet tissue. To measure the amount of FFA in the liver 100 mg wet liver was homogenized in 0.9 mL saline and centrifuged at 3000 rpm for 15 minutes and the suspension was collected for the determination of FFA content by using commercial kits (Nanjing Jiancheng Institute of Biotechnology, Nanjing, China) according to the manufacturer's instructions.

### 2.6. Serum Biochemical Parameter Analysis

The analysis of serum including TG, FFA, low-density lipoprotein cholesterol (LDL-C), high-density lipoprotein cholesterol (HDL-C), alanine aminotransferase (ALT), and aspartate aminotransferase (AST) was measured by commercial kits (Nanjing Jiancheng Institute of Biotechnology, Nanjing, China) according to the manufacturer's instructions.

### 2.7. Histological Examination and Assessment

Sections of the liver samples (4 *μ*m thick) or the frozen liver tissues (5 *μ*m thick) were stained with hematoxylin-eosin (H&E) or oil red O and were examined under light microscope (Olympus Medical Systems Corp, Tokyo, Japan). Disease activity was assessed with the use of the nonalcoholic fatty liver disease activity score, which is based on a standardized grading system for steatosis (on a scale of 0 to 3), lobular inflammation (on a scale of 0 to 3), and hepatocellular ballooning (on a scale of 0 to 2), with the higher score indicating as increasing severity [16]. 

### 2.8. Preparation of QHD-Containing Serum

Twenty male S.D. rats, weighing 170 ± 20 g, were randomly divided into two groups, QHD-containing serum group and vehicle control group. QHD-containing serum group rats were gavaged with intragastric QHD twice a day for 3 consecutive days (0.5 mL/100 g body weight/time), and vehicle control group rats were gavaged with intragastric deionized water. Blood was collected 1 h after the last administration via abdominal aorta and then centrifuged. Serum of the same group was pooled, filtered through 0.22 *μ*mol/L filter, inactivated at 56°C for 30 minutes, split, and stored at –70°C [17].

### 2.9. Cell Culturing

Human hepatocyte L02 cells (Institutes of Biochemistry and Cell Biology, Shanghai Institutes for Biological Sciences, CAS, Shanghai, China) were cultured in RPMI medium 1640 (GIBCO Invitrogen Corporation, Carlsbad, CA, USA) containing 10% fetal bovine serum (Shisheng cell biology technique Co. Ltd., Shanghai, China) with 5% CO_2_ in a cell culture incubator (Heraeus Holding GmbH, Germany) at 37°C.

### 2.10. Hepatocyte Steatosis Model Conditions

To determine the appropriate incubating time and concentration of long-chain FFA to duplicate hepatocyte steatosis model, L02 cells were incubated with or without three different concentrations of FFA (oleate/palmitate, 1/0.5, 0.5/0.25, and 0.25/0.125 mM/mM) (Sigma, St. Louis, MO, USA) in a culture medium containing 1% bovine serum albumin. At the time points of 6, 12, 24, and 48 h, cells were collected and the content of TG was determined by using standard kit.

### 2.11. Cell Viability Assay

Cell viability was determined by the alamaraclue assay. L02 cells were seeded at a density of 3,000 cells per well in 96-well cell culture plates. After cells grown in a single layer in the plates under the normal growth conditions, the medium was replaced with RPMI-1640 containing 5%, 10%, and 15% vehicle control or QHD serum for 48 h. Then 10% alamarBlue (Sigma, St. Louis, MO, USA) solution was added to the medium for 4 h. As a negative control, alamarBlue was added to the medium in the well without cells. After the medium was removed, isopropanol was added and the absorbance was read at 570 nm by using a microplate reader (Molecular Devices, Sunnyvale, CA, USA). The OD values were compared to determine whether the QHD-containing serum has an effect on cell viability.

### 2.12. *In Vitro* Experimental Design

After culturing for 24–48 h, L02 cells were divided into 4 groups as control group, FFA group, FFA + 5% QHD-containing serum group, and FFA + 10% QHD-containing serum group. To make sure the difference in the effects of QHD-containing serum is from QHD and not from serum, vehicle control was used. 5% and 10% vehicle control serum were added to control group and FFA group, while 5% and 10% QHD-containing serum were added to FFA + 5% QHD-containing serum group and FFA + 10% QHD-containing serum group, respectively. Meanwhile cultured with the corresponding serums for 24 h, cells in FFA group, FFA + 5% QHD-containing serum group, and FFA + 10% QHD-containing serum group were treated with FFA (0.5 mM oleate/0.25 mM palmitate) for 24 h.

### 2.13. Measurement of Cellular TG Content

Total lipids in the cells and the medium were extracted and purified by using the method from Heider JG [18]. The content of TG was determined with a biochemistry assay kit (Dongou Biology Technique Co. Ltd., Zhejiang, China), according to the manufacturer's instructions, and expressed as mg/g prot. 

### 2.14. Cell Oil Red O Staining

The cells were washed with PBS twice, fixed with 10% formalin at room temperature for 10 min, and stained with oil red O (Sigma, St. Louis, MO,USA) at 60°C for 10 min. The cells were observed in an Olympus (Olympus Corporation, Tokyo, Japan) microscope and documented.

### 2.15. Preparation of Hepatic and Cell Nuclear Fractions

Nuclear fractions of liver tissue and L02 cells were prepared with commercial cytoplasmic and nuclear protein extraction kits (Fermentas Inc., N/A, Canada) according to the manufacturer's instructions.

### 2.16. Western Blot Analysis

Liver tissue and L02 cells were homogenized in a lysis buffer (150 mM NaCl, 1% Nonidet P-40, 0.1% SDS, 50 mM Tris-HCl pH7.4, 1 mM EDTA, 1 mM PMSF, 1x Roche complete miniprotease inhibitor cocktail, and 1x Roche PhosSTOP phosphatase inhibitor cocktail). The supernatants were collected after centrifugation at 10,000 g at 4°C for 15 min. Protein concentration was determined by using a BCA protein assay kit (Beyotime Institute of Biotechnology, Jiangsu, China). Equal amounts of protein were separated by 10% SDS gel electrophoresis (SDS-PAGE) under denaturing and nonreducing conditions and transferred to a nitrocellulose membrane. The membrane was blocked with 5% nonfat milk in TBST at room temperature for 1 h and then incubated with primary antibody at 4°C overnight. After thrice washing in TBST, the blots were incubated with horseradish-coupled secondary antibody. The signals were visualized using enhanced chemical luminescent (ECL) system (Pierce Biotechnology, Inc., Rockford, IL, USA) and recorded in the X-ray film.

### 2.17. Statistical Analysis

Data are expressed as mean ± standard deviation. Data were analyzed by using a one-way analysis of variance as well as the least significant difference test, and *P* < 0.05 was considered statistically significant.

## 3. Results

### 3.1. HPLC Analysis of QHD

**Figure 1:**
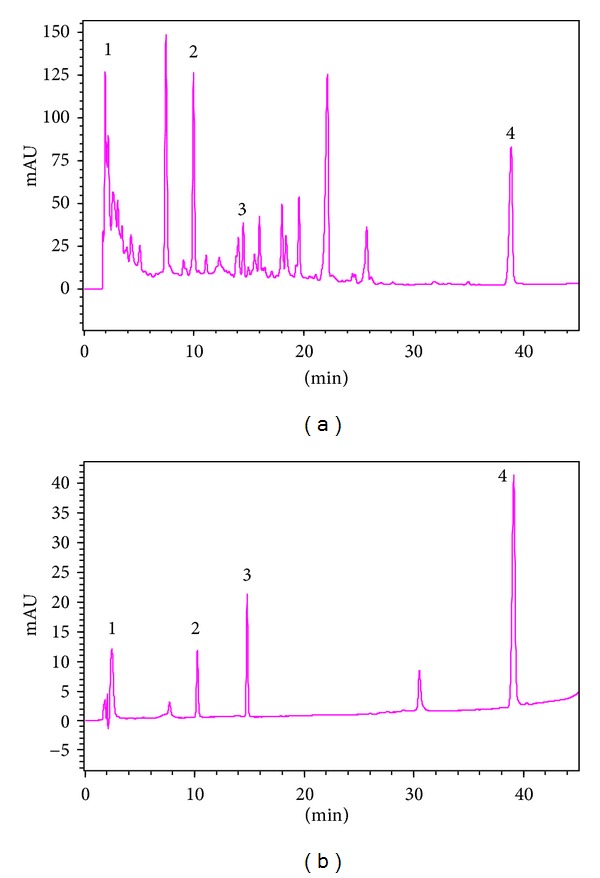
The HPLC chromatogram of QHD. (a) QHD. (b) Standard compounds. Compounds were labeled as internal standard, chlorogenic acid (2.457 min, Peak 1), polygonin (10.213 min, Peak 2), resveratrol (14.792 min, Peak 3), and jasminoidin (39.104 min, Peak 4).

The HPLC chromatogram showed that chlorogenic acid, polygonin, resveratrol, and jasminoidin in QHD were well resolved by gradient elution (Figure 1). Validation of this assay method indicated that all coefficients of intraday and inter-day variation were lesser than 10%, and the relative errors were below 20%. We selectively detected polygonin and jasminoidin contents in QHD. Analysis showed that 1 g QHD contained 1.77 mg of polygonin and 2.14 mg of jasminoidin.

### 3.2. Effects of QHD on Body Weight and Food Intake of HFD-Fed Rats

As shown in Table 2, there was no significant difference in the initial body weight between the 3 groups. At the end of THE 8th week, HFD group rats weighed significantly higher than the NC group (*P* < 0.01). The HFD + QHD group rats gained lesser weight than those in the HFD group, although there was no significant difference between them. No significant difference in daily food intake amount was observed between the 3 groups during the experimental period.

### 3.3. Effects of QHD on Hepatic Lipids of HFD-Fed Rats

The hepatic TG level was significantly higher in the HFD group rats than in the ND group rats (Table 2, *P* < 0.01). In QHD treatment in HFD + QHD group rats the hepatic TG levels were reduced by 46.7% (*P* < 0.01). Similarly, QHD treatment also produced a significant reduction in hepatic FFA concentration when compared to HFD group (Table 2). The data showed that QHD treatment ameliorated hepatic steatosis significantly.

### 3.4. Effects of QHD on Serum Lipids, ALT, and AST Activities of HFD-Fed Rats

At the end of administration, compared with the ND group rats, serum TG, FFA, and LDL-C levels significantly increased and serum HDL-C level was reduced in the HFD group rats (*P* < 0.05 or *P* < 0.01). QHD intake showed lowered LDL-C levels when compared with the HFD group (*P* < 0.01). There was no notable difference in serum TG, FFA, and HDL-C between the HFD + QHD and HFD groups rats (Table 2). Compared with the ND group rats, serum ALT, AST activities significantly increased in HDF group (*P* < 0.01). QHD intake showed lowered ALT, AST levels when compared with the HFD group (*P* < 0.05 or *P* < 0.01) (Table 2), suggesting that QHD could protect the liver injury induced by the HFD feeding.

### 3.5. Effects of QHD on Liver Morphology and Histopathology

**Figure 2:**
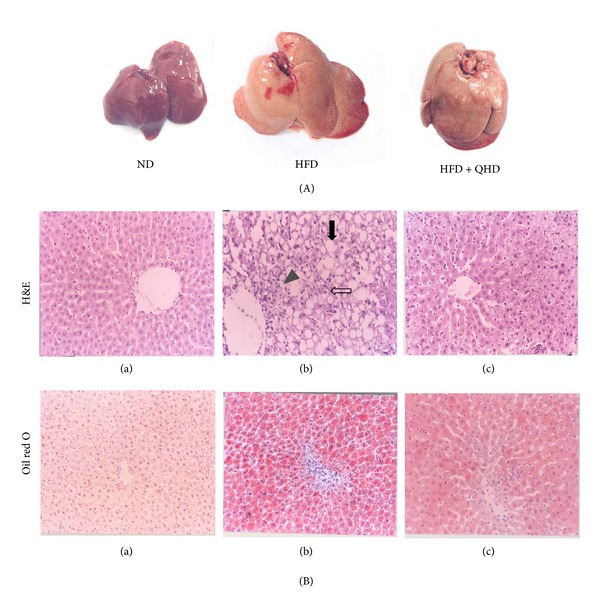
Effect of QHD on hepatic morphology and pathological changes. (A) The liver samples of ND, HDF, and HFD + QHD rats. (B) Histological micrograph of liver specimen from ND, HDF, and HFD + QHD rats (H&E and oil red O staining, magnification ×200). (a) The ND group; (b) the HFD group; (c) the HFD + QHD group. The major histopathological change induced by HFD in rat liver was hepatocyte steatosis (filled arrow) with inflammation (arrowhead) and ballooning (open arrow).

The livers of HFD group rats showed significant increase in volume, tawny, greasy, and brittle. Livers of HFD + QHD group rats gained less volume, rosier and felt more flexible than those in the HFD group (Figure 2(A)).

The histological changes in livers were examined in H&E and oil red O stained sections. The liver samples of HFD rats showed degeneration of hepatocytes with abundant fat deposition, mononuclear inflammatory cells infiltration, and hepatocyte ballooning (Figure 2(B)). However, alleviated fatty degeneration, inflammation, and hepatocyte ballooning were observed in HFD + QHD group rats (*P* < 0.05) (Table 3). Histological examination with oil red O staining showed that QHD treated rats had significantly less fat deposition in hepatocytes when compared to HFD group rats (Figure 2(B)). 

### 3.6. Effects of QHD on AMPK and ACC Activation in Hepatic Tissues of HFD-Fed Rats

**Figure 3:**
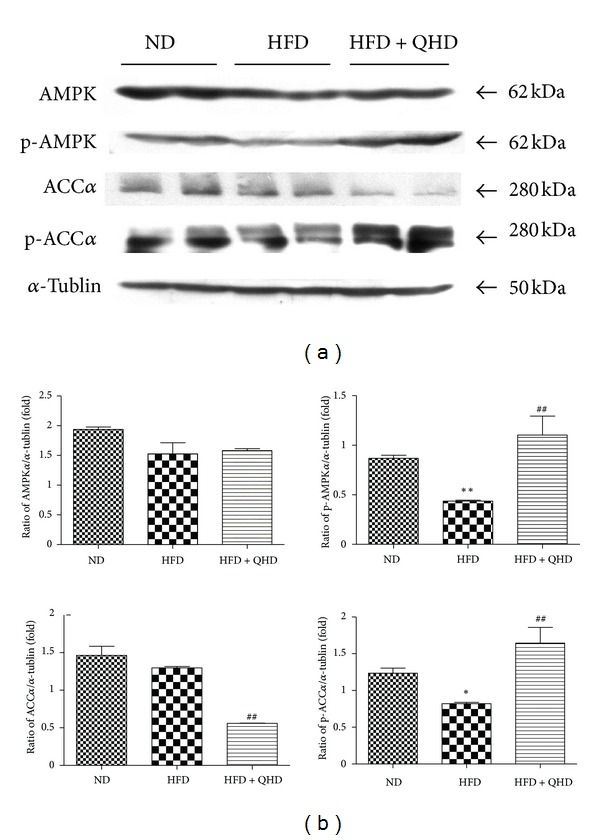
Effects of QHD on AMPK and ACC*α* protein expression and activation in hepatic tissues of HFD-fed rats. (a) Western blot. (b) Gray-level score. **P < 0.01, versus the ND group; *P < 0.05, versus the ND group; ^##^ *P* < 0.01, versus the HFD group.

AMPK is a key regulator of fatty acid oxidation and lipogenesis in metabolic tissues. Activated AMPK switches on catabolic pathways that produce ATP, primarily by increasing fatty acid oxidation and inhibiting lipid synthesis [8]. To determine whether the effect of QHD on hepatic lipid accumulation is mediated by AMPK activation, we first determined the Thr-172 phosphorylation of AMPK because this is an essential marker of AMPK activity. As shown in Figure 3, there was no difference in the expression of endogenous total AMPK protein between ND, HFD + QHD, and HFD groups. However, HFD-fed rats showed significant reduction in AMPK phosphorylation and QHD significantly stimulated AMPK phosphorylation (Figure 3).

To determine whether AMPK activation affects its downstream target genes, we examined the activity of ACC, a key enzyme involved in the regulation of fatty acid metabolism. Although HFD-fed did not change endogenous total ACC*α* protein expression, QHD suppressed endogenous total ACC*α* protein expression significantly. Ser79 phosphorylation of ACC*α* in HFD-fed rats group rats was significantly lower than that in ND group rats and QHD also increased ACC*α* phosphorylation significantly (Figure 3).

### 3.7. Effects of QHD on Total or Nuclear SREBP-1 and ChREBP Protein Expression in Hepatic Tissues of HFD-Fed Rats

Lipogenesis is transcriptionally mediated by the transcription factors, SREBP-1 and ChREBP. ChREBP is phosphorylated by activated AMPK, which inhibits its entry into the nuclear [10]. SREBP-1c expression is reduced by activated AMPK through undefined mechanisms. When AMPK is activated, it results in phosphorylation and inhibition of both ACC and ChREBP as well as a reduction in the expression levels of SREBP-1c [10]. To determine whether QHD affects the two transcription factors, SREBP-1 and ChREBP, we examined the hepatic total and nuclear protein levels. 

**Figure 4:**
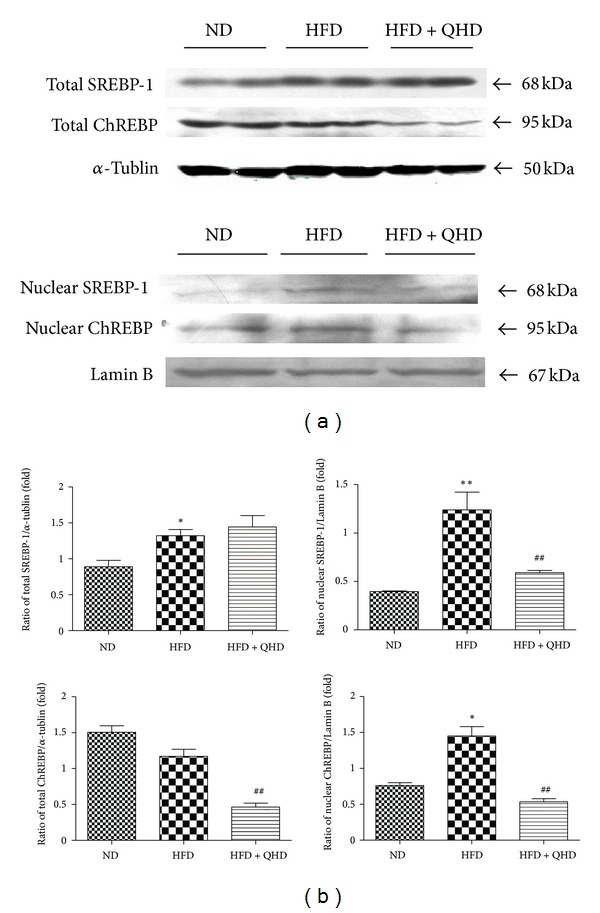
Effects of QHD on SREBP-1 and ChREBP in total and nuclear protein expressions in hepatic tissuesof HFD-fed rats. (a) Western blot. (b) Gray-level score. **P < 0.01, versus the ND group; *P < 0.05, versus the ND group; ^##^ *P* < 0.01, versus the HFD group.

The hepatic total SREBP-1 in HFD group rats was significantly higher than that in ND group rats (*P* < 0.05), but there was no change between HFD group and HFD + QHD group (Figure 4). The nuclear protein expression of SREBP-1 in HFD group rats was significantly higher in ND group rats, but it was decreased in HFD + QHD group rats when compared to HFD group rats.

Although the hepatic total ChREBP protein in HFD group rats showed no significant change on comparison to ND-fed rats, the total ChREBP protein in HFD + QHD group rats was significantly lower than that in HFD group rats (Figure 4). The nuclear protein expression of ChREBP in HFD group rats was significantly higher than that in ND group rats. The protein levels of hepatic nuclear ChREBP were decreased significantly in HFD + QHD group rats. 

### 3.8. Exploring the L02 Cells Steatosis Model Conditions and Effects of QHD-Containing Serum on Cell Viability

**Figure 5:**
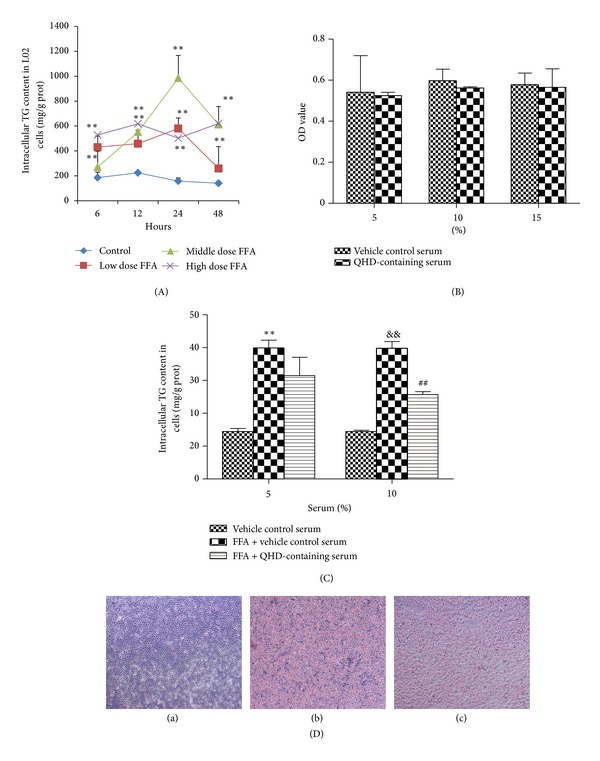
Effects of QHD-containing serum on cellular steatosis in L02 cells stimulated with FFA. (A) Intracellular TG content in L02 cells incubated with different concentrations of FFA for different times. The concentrations of low, middle, and high dose of FFA were 0.25 mM oleate/0.125 mM palmitate, 0.5 mM oleate/0.25 mM palmitate, and 1 mM oleate/0.5 mM palmitate, respectively. **P < 0.01, versus the control group at the same time point. (B) Effects of QHD-containing serum on cell viability of L02 cells. Cell viability was determined by the alamarBlue assay. (C) Effects of different concentrations of QHD-containing serum on intracellular TG contents in L02 cells incubated with FFA. **P < 0.01, versus the 5% vehicle control group; ^&&^ *P* < 0.01, versus the 10% vehicle control group; ^##^ *P* < 0.01, versus the FFA + 10% vehicle control group. (D) Effect of QHD-containing serum on cellular lipid droplets in L02 cells stimulated with FFA (oil red O staining, magnification ×100). (a) Control group; (b) FFA group; (c) FFA + QHD group.

To determine the appropriate conditions to duplicate hepatocyte steatosis model, L02 cells were incubated with or without oleate/palmitate of different concentrations for 6, 12, 24, and 48 h, and then the cell TG contents were detected. As shown in Figure 5(A), after being incubated with middle dose of FFA for 24 h, the TG content of the cells was at the highest. So the optimal condition of molding hepatocyte steatosis model was that the cells would be incubated with FFA at the concentration of 0.5 mM oleate/0.25 mM palmitate for 24 h.

Cell viability was determined by the alamarBlue assay. The OD value of vehicle control serum was compared with QHD-containing serum treated cells at different concentrationS to determine whether the QHD-containing serum had any effect on cell viability. The result showed that incubation with 5%, 10%, and 15% QHD-containing serum for 24 h had no significant change (*P* > 0.05) in cell viability (Figure 5(B)).

### 3.9. Effect of QHD-Containing Serum on TG Content and Cellular Lipid Droplets in L02 Cells Stimulated with FFA

After being stimulated with FFA and incubated with it for 24 h, the cellular TG contents were increased significantly. QHD-containing serum of 5% had no significant effect on the cellular TG content when compared with 5% vehicle control serum, but 10% QHD-containing serum decreased the cellular TG content significantly (*P* < 0.05) when compared with 10% vehicle control serum (Figure 5(C)). So 10% QHD-containing serum was adopted in the following pharmacological experiments.

L02 cells were stained by oil red O after being incubated with FFA and corresponding serum for 24 h. As shown in Figure 5(D), lipid droplets in cells of FFA + 10% vehicle control serum group (FFA) were significantly more than those in vehicle control serum group (Control). But in FFA + 10% QHD-containing serum group (FFA + QHD) there were much lesser lipid droplets than in FFA group.

### 3.10. Effects of QHD-Containing Serum on AMPK and ACC*α* Activation in L02 Cells Stimulated with FFA

**Figure 6:**
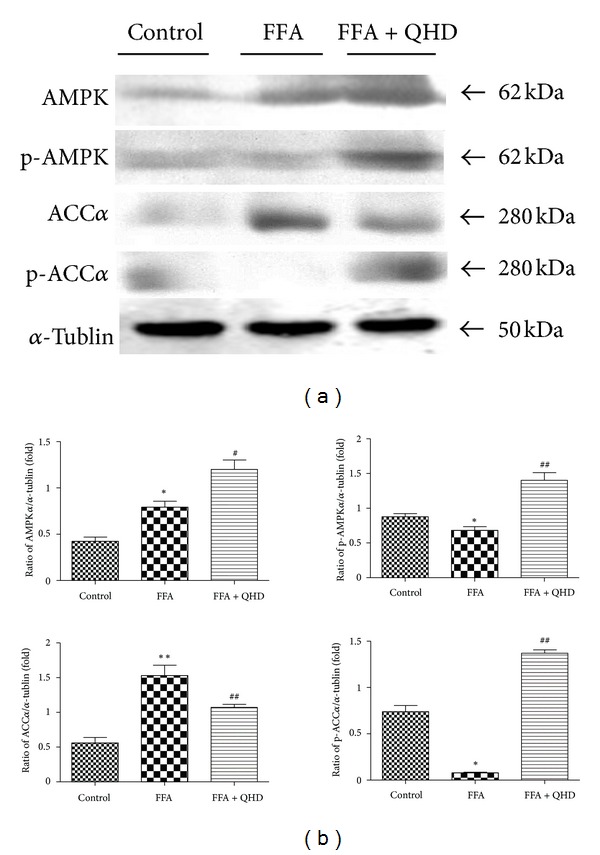
Effects of QHD on SREBP-1 and ChREBP in total and nuclear protein expressions in L02 cells stimulated with FFA. (a) Western blot. (b) Gray-level score. **P < 0.01, versus the control group; ^##^ *P* < 0.01, versus the FFA group; ^#^ *P* < 0.05, versus the FFA group.

As shown in Figure 6, the expression of AMPK was higher after being stimulated with FFA; QHD-containing serum could increase AMPK expression. AMPK phosphorylation in L02 cells was decreased after being stimulated with FFA for 24 h, but QHD-containing serum significantly stimulated its phosphorylation.

Then we observed effect of QHD-containing serum on ACC*α* activation. As shown in Figure 6, FFA significantly upregulated the ACC*α* expression and downregulated the phosphorylation of ACC*α*. QHD-containing serum significantly attenuated the upregulation of ACC*α* and the downregulation of ACC*α* phosphorylation.

### 3.11. Effects of QHD-Containing Serum on SREBP-1 and ChREBP Protein Expression in L02 Cells Stimulated with FFA

**Figure 7:**
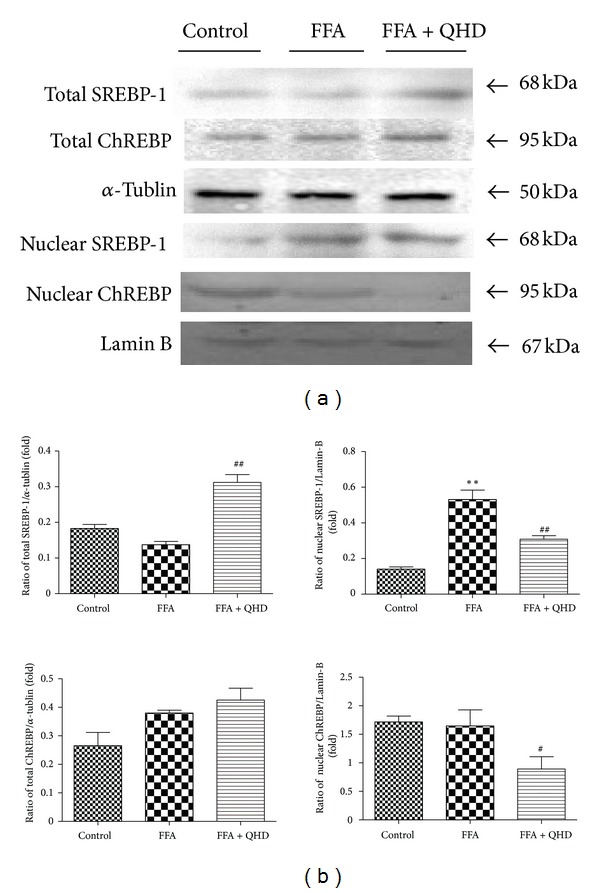
Effects of QHD on AMPK and ACC*α* protein expression and activation in L02 cells stimulated with FFA. (a) Western blot. (b) Gray-level score. **P < 0.01, versus the control group; *P < 0.05, versus the control group; ^##^ *P* < 0.01, versus the FFA group; ^#^ *P* < 0.05, versus the FFA group.

To determine whether AMPK activation affects SREBP-1 and ChREBP, we examined the protein levels of them both in hepatocyte total and nuclear protein. FFA had no obvious effect on total protein expression of SREBP-1, but it significantly upregulated the nuclear protein expression of SREBP-1. QHD-containing serum could significantly downregulate the nuclear protein expression and upregulate the total protein expression of SREBP-1 in L02 cells stimulated with FFA (see Figure 7).

Though both total and nuclear protein expressions of ChREBP had no significant change after being stimulated with FFA, QHD-containing serum could significantly down-regulate the nuclear ChREBP protein expression in L02 cells stimulated with FFA (see Figure 7).

## 4. Discussion

Our present study showed that the administration of QHD has preventive effects against hepatic lipid accumulation* in vivo* and *in vitro* by activation of AMPK signaling.

In China, QHD has been used in clinical practice to alleviate NAFLD [13]. Previous animal studies showed that QHD could ameliorate dyslipidemia and hepatic steatosis [14, 15]. However, the underlying molecular mechanism needs to be further investigated. AMPK activity is recognized as a major regulator of liver and whole body lipid homeostasis. Many herbal medicines have been shown to prevent and treat obesity and associated metabolic disorders by regulating AMPK activity, such as resveratrol, (−)-epigallocatechin-3-gallate, and berberine, et al. [19–21]. Considering the key role of AMPK activation in regulating lipid metabolism, we hypothesized that AMPK may play a key role on the effects of QHD against hepatic lipid accumulation. So, in this study, we observed the effect of QHD on AMPK activation and the related pathway in fatty acid metabolism both *in vitro *and *in vivo*.

We used a high-fat diet for 8 weeks to duplicate fatty liver model in rats. The rats in HFD groups had higher body weight, liver TG and FFA levels, serum TG and LDL-C levels, and serum ALT, AST levels and lower HDL-C level compared to rats in normal control group. These results indicated that the fatty liver model with triglycerides accumulation in the liver was induced successfully by the high-fat diet. Using these model rats, we demonstrated that 4-week QHD treatment ameliorated hepatic steatosis and decreased the accumulation of TG and FFA in the liver. This was shown histologically and biochemically. In addition, elevated serum level of LDL-c was suppressed by QHD administration. In the HFD group, the activities of liver function markers, including serum ALT and AST, were significantly elevated relative to those in the ND group and were improved by QHD supplementation. The decrease of serum ALT and AST by QHD may account for the improvement of the liver histology and less fat infiltration of hepatocytes. The results indicate that the administration of QHD can dramatically suppress the development of HFD-induced fatty liver. Moreover, QHD supplementation reversed the HFD-induced decrease in the phosphorylation levels of AMPK and ACC, which are related to lipogenesis. Also, QHD decreased hepatic nuclear protein expression of SREBP-1 and ChREBP in liver, which are two key transcription factors regulating *de novo *lipogenesis.

In order to study further mechanism improving hepatic lipid metabolism *in vitro*, L02 cells, a kind of human hepatocyte, were incubated with a mixture of FFA (2 : 1 oleate/palmitate) to develope cellular steatosis. FFA acts as a potent cell toxin and induces the overstorage of lipids in the nonfatty tissue. The abnormal increase in FFA plays an important role in the pathogenesis of NAFLD [22, 23]. We used three different doses of FFA to stimulate L02 cells for 6, 12, 24, and 48 h and found that being incubated with 0.5 mM oleate/0.25 mM palmitate for 24 h were appropriate conditions to establish cellular steatosis model. 

Chinese composite recipe aims at multitarget in human body and includes many different materials and chemical compositions. After Chinese composite recipe is taken, metabolism will happen in stomach, intestine, and liver and the effect of Chinese composite recipe will play a role [17]. Therefore, for *in vitro* experiment to reproduce the features of QHD after metabolism in digestive system, we prepared QHD-containing serum. Then, we confirmed that the doses of 5% and 10% (vol/vol) used in this study are safe based on the MTT assay* in vitro*.

With the hepatocytes steatosis model, we observed that the 10% QHD-containing serum could inhibit cellular TG content and alleviate cellular fatty drops significantly. The study demonstrated that QHD might have a direct effect on hepatocellular lipid metabolism. The same as the results in the animal study, QHD supplementation reversed the FFA-induced decrease in the phosphorylation levels of AMPK and ACC and decreased hepatic nuclear protein expression of SREBP-1 and ChREBP in steatosis L02 cells.

**Figure 8:**
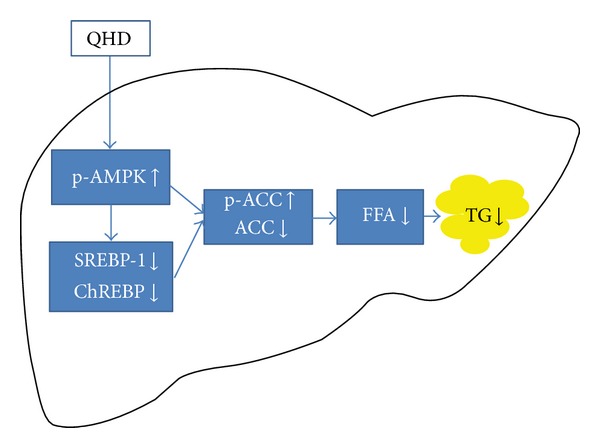
QHD inhibits hepatic lipid accumulation by activating AMPK. QHD significantly stimulates AMPK phosphorylation, decreases nuclear SREBP-1 and ChREBP protein contents, down-regulates ACC*α* expression and up-regulates phosphorylation of ACC*α*, and then decreases hepatic *de novo *lipogenesis and accumulation.

As shown in the results of HPLC, chlorogenic acid, polygonin, resveratrol, and jasminoidin are major constituents in QHD. Resveratrol (3,5,4′-*trans*-trihydroxystilbene) is a naturally occurring phytoalexin that is found in many medicinal plants, such as *polygonum cuspidatum Siebetold and Zucc*, grape skin, peanuts, and red wine. Resveratrol exhibits remarkable biological activities in metabolic diseases, including increased insulin sensitivity, reduced insulin-like growth factor-1 (IGF-I) levels, increased AMPK and peroxisome proliferator-activated receptor-*γ* coactivator 1*α* (PGC-1*α*) activity, increased mitochondrial number, and improved motor function [24–26]. Chlorogenic acid, a major constituent in *Artemisia capillaries* and green coffee bean et al.,has been shown to be an effective nutraceutical in reducing weight in preobese adults and may be an inexpensive means of preventing obesity in overweight adults [27]. Chlorogenic acid has a significant influence on glucose metabolism, which was well demonstrated by Kojima et al. [28], as they were able to demonstrate a significant improvement in glucose tolerance in Zucker rats. Ong et al. [29] reported that chlorogenic acid stimulates glucose transport in skeletal muscle via AMPK activation. Geniposide, which is iridoid glycoside from the fruit of *Gardenia jasminoides *Ellis, is recognized as being useful against hyperlipidemia and fatty liver [30]. It has an anti-obesity effect, an insulin resistance-alleviating effect, and an abnormal lipid metabolism-alleviating effect. And the metabolite genipin shows a direct effect on the liver, inducing suppressing the intracellular lipid accumulation caused by the free fatty acid treatment and also significantly increasing the intracellular expression of peroxisomal proliferator-activated receptor (PPAR*α*) [28]. From these, we presume that chlorogenic acid, resveratrol, and jasminoidin might be major contributors to the beneficial effects of QHD on hepatic lipid accumulation by activating AMPK (Figure 8).

## 5. Conclusion

In conclusion, our studies suggest that QHD could inhibit hepatic lipid accumulation by activating AMPK *in vivo* and *in vitro*. QHD significantly stimulates AMPK phosphorylation, decreases nuclear SREBP-1 and ChREBP protein contents, downregulates ACC*α* expression and upregulates phosphorylation of ACC*α*, and then decreases hepatic *de novo *lipogenesis and accumulation. Our findings might be a valuable resource in supporting Qushi Huayu Decoction to be used in the clinical purpose.

## Figures and Tables

**Table 1:** Different components in the formula of QHD.

Chinese name	Pharmaceutical name	Family name	Production place	Processing method
Yin Chen	*Artemisia capillaries* Thunb. (above–ground parts, dried)	Compositae	Anhui Province, China	Ethanol extraction
Hu Zhang	*Polygonum cuspidatum* Sieb. *et* Zucc. (*rhizome, root, and dried*)	Polygonaceae	Jiangsu Province, China	Ethanol extraction
Jiang Huang	*Curcuma longa* L. (*rhizome, root, dried*)	Zingiberaceae	Sichuan Province, China	Ethanol extraction
Tian Ji Huang	*Hypericum japonicum * Thunb. (*whole plant, dried*)	Clusiaceae	Jiangxi Province, China	Water extraction
Zhi Zi	*Gardenia jasminoides *Ellis. (*ripe fruit,dried*)	Rubiaceae	Fujian Province, China	Water extraction

**Table 2:** Body weight, food intake, and biochemical parameters in rats in different study groups.

Parameter	ND (*n* = 10)	HFD (*n* = 10)	HFD + QHD (*n* = 10)
Body weight before treatment (g)	168.7 ± 6.9	170.2 ± 5.4	171.1 ± 6.3
Body weight after treatment (g)	375.3 ± 19.9	411.9 ± 33.4**	392.6 ± 32.9
Food intake (g rat^−1^ per day)	19.8 ± 2.3	22.1 ± 2.5	21.6 ± 2.1
Liver			
TG (mg/g)	11.4 ± 6.4	54.7 ± 20.3**	29.2 ± 11.7^##^
FFA (*μ*mol/g prot)	475 ± 178	786 ± 174**	518 ± 139^#^
Serum			
TG (mmol/L)	0.23 ± 0.06	0.31 ± 0.09**	0.26 ± 0.05
FFA (mmol/L)	0.53 ± 0.10	0.63 ± 0.09*	0.62 ± 0.07
HDL-C (mmol/L)	0.41 ± 0.05	0.34 ± 0.07**	0.37 ± 0.09
LDL-C (mmol/L)	0.65 ± 0.18	1.11 ± 0.27**	0.85 ± 0.10^##^
ALT (U/L)	31.8 ± 6.5	67.9 ± 16.2**	44.3 ± 15^#^
AST (U/L)	80.7 ± 15.8	135.4 ± 15**	52.1 ± 21.4^##^

Quantitative data are expressed as mean ± SD. Statistical analysis of the data for multiple comparisons was performed by ANOVA.

*P < 0.05, versus the ND group.

**P < 0.01, versus the ND group.

^#^ *P* < 0.05, versus the HFD group.

^##^ *P* < 0.01, versus the HFD group.

**Table 3:** Average score of histopathological findings in livers.

Parameter	ND (*n* = 10)	HFD (*n* = 10)	HFD + QHD (*n* = 10)
Steatosis	0 ± 0	2.6 ± 0.54**	1.5 ± 0.71^##^
Inflammation	0 ± 0	2.0 ± 0.63**	1.5 ± 0.48^#^
Ballooning	0 ± 0	2.0 ± 0**	1.25 ± 0.46^#^

Quantitative data are expressed as mean ± SD. Statistical analysis of the data for multiple comparisons was performed by one-way ANOVA.

**P < 0.01, versus the ND group.

^#^ *P* < 0.05, versus the HFD group.

^##^ *P* < 0.01, versus the HFD group.

## Abbreviations

ACC: Acetyl-CoA carboxylase
ALT: Alanine aminotransferase
AMPK: AMP-activated protein kinase
AST: Aspartate aminotransferase
ChREBP: Carbohydrate response element-binding protein
FFA: Free fatty acid
HDL-C: High-density lipoprotein cholesterol
HPLC: High-pressure liquid chromatography
LDL-C: Low-density lipoprotein cholesterol
NAFL: Nonalcoholic fatty liver
NAFLD: Nonalcoholic fatty liver disease
NASH: Nonalcoholic steatohepatitis
SREBP-1: Sterol regulatory element-binding protein-1
TG: Triglyceride
TNF-*α*: Tumor necrosis factor *α*.
